# Differences in detected safety signals between benzodiazepines and non-benzodiazepine hypnotics: pharmacovigilance study using a spontaneous reporting system

**DOI:** 10.7150/ijms.51658

**Published:** 2021-01-01

**Authors:** Manabu Toyoshima, Yoshihiro Noguchi, Manami Otsubo, Tomoya Tachi, Hitomi Teramachi

**Affiliations:** 1 Department of Pharmacy, Kawasaki rinko general hospital; 3-13-1 Nakashima, Kawasaki-ku, Kawasaki, 210-0806, Kanagawa, Japan; 2 Laboratory of Clinical Pharmacy, Gifu Pharmaceutical University; 1-25-4, Daigakunishi, Gifu, 501-1196, Gifu, Japan; 3 Department of Pharmacy, Gifu University Hospital; 1-1, Yanagido, Gifu, 501-1194, Gifu, Japan; 4 Laboratory of Community Healthcare Pharmacy, Gifu Pharmaceutical University; 1-25-4, Daigakunishi, Gifu, 501-1196, Gifu, Japan

**Keywords:** Japanese Adverse Drug Event Report database, signal detection, non-benzodiazepine-based hypnotics, benzodiazepine-based hypnotics

## Abstract

**Introduction:** In recent years, there has been an increasing number of people who feel sleep-deprived owing to sudden changes in the social environment. Patients prescribed benzodiazepine-based hypnotics (BZ drugs) also develop movement disorder action and memory disorders as adverse events (AEs), and they have further problems such as dependency and tolerance because of long-term use. Therefore, the use of non-benzodiazepine-based hypnotics (Z-drugs) is recommended for patients with insomnia. However, as AEs have also been reported for Z-drugs, it is important to identify these when switching hypnotics.

**Methods:** To understand AEs to be noted when switching from BZ drugs to Z-drugs, we evaluated the differences in AEs developed by both these drugs using volcano plots and safety signals. For this, data registered in the Japanese Adverse Drug Event Report database were used.

**Results:** The volcano plot and safety signals revealed six characteristic Z-drug-induced AEs. Parasomnias (ln odds ratio [*OR*]: 3.28, -log *P*: 4.34, proportional reporting ratio [*PRR*]: 23.47, *χ*^2^: 309.27), Cortical dysfunction NEC (ln *OR*: 2.76, -log *P*: 4.34, *PRR*: 3.62, *χ*^2^: 16.14), and Psychiatric symptoms NEC (ln *OR*: 2.66, -log *P*: 2.18, *PRR*: 2.51, *χ*^2^: 6.63) were detected only in Z-drugs, and safety signals of Suicidal and self-injurious behaviour, Deliria, and Overdoses NEC were also detected with BZ drugs. However, the strength of safety signals was much higher with the Z-drugs.

**Conclusion:** AEs related to falls and bone fractures are expected to be more strongly onset in BZ drugs than in Z-drugs, which are said to have less muscle relaxant action. However, there was no particularly significant difference in this parameter between the two drug classes. Understanding the difference between these AEs of Z-drugs and BZ drugs is important for the proper use of hypnotics.

## Introduction

In recent years, the number of people who experience sleep deprivation owing to a sudden change in the social environment is increasing, and 4% to 6% of Japanese adults routinely use hypnotics 1. In particular, the number of benzodiazepine-based hypnotics (BZ drugs) used in Japan is higher than that used overseas.

BZ drugs act on benzodiazepine receptors in the brain and exert hypnotic action by enhancing the activity of the γ-aminobutyric acid (GABA) system, which has an inhibitory potential on the central nervous system. BZ drugs are less likely to cause fatal damage owing to the suppression of the respiratory center compared with barbiturates, which act on the same GABA receptor and are widely used in clinical practice 2. The use of BZ drugs also leads to adverse events (AEs) such as movement disorders and memory disorders; furthermore, their long-term use is associated with problems such as dependency and tolerance. Therefore, the “Clinical Guidelines for Proper Use of Hypnotics and Drug Withdrawal” of The Japanese Society of Sleep Research state that the quality of evidence is “moderate” and the recommendation is “weak.” It recommends the use of non-benzodiazepine-based hypnotics (Z-drugs) for patients who experience sleeplessness 2. However, as AEs have also been reported for Z-drugs, it is important to identify these when switching hypnotic drugs.

Safety signals based on the principle of disproportionality in the difference between the ratio of AEs reported is used as an index of detection. Safety signals can detect unknown AEs at an early stage, and numerous risk assessments based on this have been reported 3-5. There are several algorithms for signal detection 6-11. Furthermore, in recent years, the use of various data mining methods has been proposed 12-15. Among them, a method of visualizing the relationship between the *OR* and the statistically significant difference between the AEs of each group when comparing reported AEs among groups is often used 12, 13. Known as the volcano plot 16, it is often used in the field of bioinformatics to visually understand the tendency of gene expression, and in recent years, it has also been utilized in the field of pharmacovigilance because it can easily evaluate the tendency of AE occurrence 12, 13. Therefore, we evaluated the differences in AEs associated with BZ drugs and Z-drugs using the volcano plot and safety signals with the data from the Japanese Adverse Drug Event Report (JADER) database.

## Methods

### Data sources

We created a dataset based on JADER database published in May 2020. The Japanese authority the Pharmaceuticals and Medical Devices Agency that owns these data does not permit its direct sharing. Therefore, we cannot make this data available and it can be accessed directly from the website: http://www.info.pmda.go.jp/fukusayoudb/CsvDownload.jsp (in Japanese only).

JADER is divided into four files and published, but they were combined based on the identification code into one dataset. This study excludes cases with age or gender deficiencies. The dataset for analysis contained 565,454 cases.

### Definitions of suspected drugs and AEs

Suspected drugs were defined as BZ and Z-drugs. However, as Z-drugs are manufactured only as short-acting type of drugs, in this study, only ultra-short acting and short-acting BZ drugs were analyzed.

AEs to be investigated were defined as all AEs caused by BZ drugs and Z-drugs registered in JADER database. An AE is registered in the JADER database as the preferred term of the Medical Dictionary for Regulatory Activities/Japanese version (MedDRA/J); version 23.0. In this study, the target AE was defined as the high-level term (HLT).

Furthermore, the number of reports was based on the number of cases and not on the number of drug-AE combinations.

### Statistical analysis

### Volcano plot

**Figure 1:**
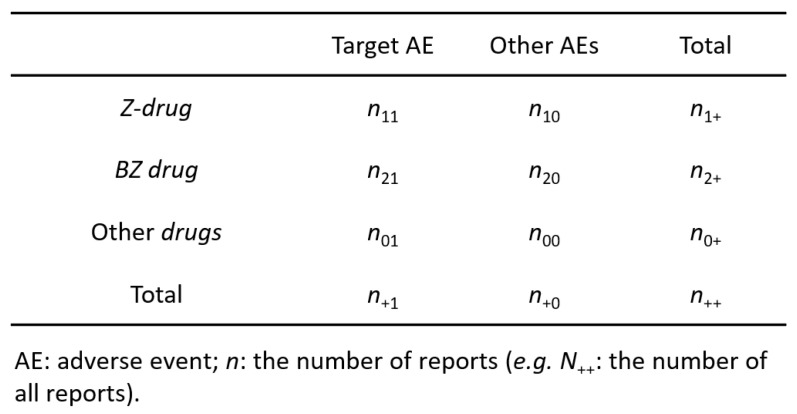
Contingency table (3 × 2) for volcano plot and signal detection

According to previous reports 12,13, we created a 3 × 2 table based on the presence or absence of Z-drugs, BZ drugs, and the target AE of each hypnotic (Figure 1). We calculated the *OR* and performed the Fisher's exact test. A volcano plot was prepared using the natural logarithm of *OR* (Eq. 1) as the abscissa and the common logarithm of *p-*value reciprocal obtained using the Fisher's exact test as the ordinate, showing the relevance of each hypnotic and AE.


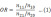


However, *OR* cannot be determined, if a 0 cell exists in *n*_11_, *n*_10_, *n*_21_, and *n*_20_, and if the frequency is low, the estimation becomes unstable. Therefore, we corrected this bias by adding 0.5 to all cells (the Haldane-Anscombe 1/2 correction).

### Signal detection

The *proportional reporting ratio* (*PRR*) 6 is a detection index used by the Medicines and Healthcare Products Regulatory Agency and the European Medicines Agency.

*PRR* was used to calculate the value of a single-drug signal. For this, a 2 × 2 contingency table was created from a 3 × 2 contingency table (Figure 1).

The following replacements were made to calculate the* PRR* and *χ*^2^*.*

For Z-drugs and all other drugs: *N*_11_ = *n*_11_, *N*_00_ = *n*_20_ + *n*_00_, *N*_10_ = *n*_10_, *N*_01_ = *n*_21_ + *n*_01_, *N*_1+_ = *n*_1+_, *N*_+1_ = *n*_+1_, *N*_0+_ = *n*_2+_ + *n*_0+_, *N*_+0_ = *n*_+0_.

For BZ drugs and all other drugs: *N*_11_ = *n*_21_, *N*_00_ = *n*_10_ + *n*_00_, *N*_10_ = *n*_20_, *N*_01_ = *n*_11_ + *n*_01_, *N*_1+_ = *n*_21+_, *N*_+1_ = *n*_+1_, *N*_0+_ = *n*_1+_ + *n*_0+_, *N*_+0_ = *n*_+0_.

Eqs. 2 and 3 were used for calculating *PRR* and *χ*^2^, respectively 6:


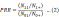



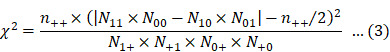


The *PRR* detection criteria were as follows: (1)* PRR* ≥ 2, (2) *χ*^2^ ≥ 4, and (3) *N*_11_ ≥ 3.

### Analysis software

The analysis software in this study used Visual Mining Studio^®^ (NTT DATA Mathematical Systems Inc. Shinjuku-ku,Tokyo, JAPAN) version 8.4 and Microsoft Excel^®^ 2019 (Microsoft Corp., Redmond, WA USA).

## Results

**Figure 2:**
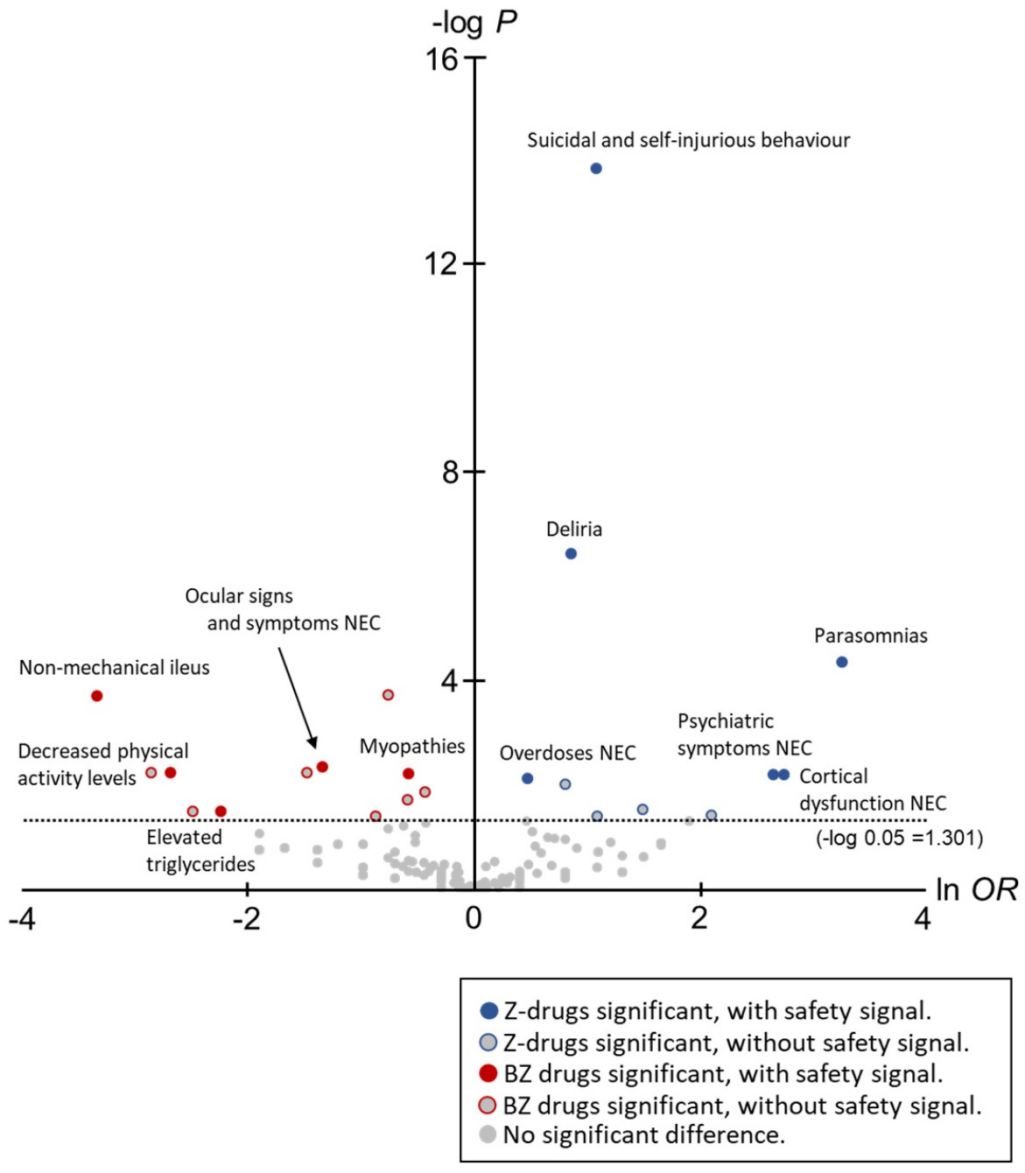
Adverse events with significant differences in onset

In this study, to comprehensively compare the trends of AEs between Z-drugs and BZ drugs, volcano plots were created and the *OR* for each AE and statistically significant differences were compared (Figure 2).

The horizontal axis in Figure 1 is ln* OR*, which means that the safety signal in the positive direction is an AE strongly related to the Z-drugs, and the safety signal in the negative direction is an AE strongly related to the BZ drugs. In contrast, the vertical axis is the -log *p*-value determined using the Fisher's exact test, suggesting that the statistically significant difference is a more pronounced AE in the positive direction.

In total, 12 AEs (HLTs) tended to occur with the onset with Z-drugs. Of these, six AEs (HLTs) were actually detected using *PRR*: Suicidal and self-injurious behaviour (ln *OR*: 1.10, -log *P*: 13.84, PRR: 16.14, χ^2^: 2743.94), Deliria (ln *OR*: 0.88, -log *P*: 6.42, *PRR*: 11.64, *χ*^2^: 1193.14), Parasomnias (ln *OR*: 3.28, -log *P*: 4.34, *PRR*: 23.47, *χ*^2^: 309.27), Cortical dysfunction NEC (ln *OR*: 2.76, -log *P*: 4.34, *PRR*: 3.62, *χ*^2^: 16.14), Psychiatric symptoms NEC (ln *OR*: 2.66, -log *P*: 2.18, *PRR*: 2.51, *χ*^2^: 6.63), and Overdoses NEC (ln *OR*: 0.49, -log *P*: 2.09, *PRR*: 23.64, *χ*^2^: 1176.82) (Figure 2, Table 2).

However, 20 AEs (HLTs) tended to occur with the onset with BZ drugs. Of these, five AEs (HLTs) were detected using *PRR*: Non-mechanical ileus (ln *OR*: -3.33, -log *P*: 3.68, *PRR*: 3.53, *χ*^2^: 15.50), Ocular signs and symptoms NEC (ln *OR*: -1.33, -log *P*: 2.32, *PRR*: 16.13, *χ*^2^: 214.17), Decreased physical activity levels (ln *OR*: -2.68, -log *P*: 2.20, *PRR*: 11.06, *χ*^2^: 34.84), Myopathies (ln *OR*: -0.57, -log *P*: 2.20, *PRR*: 2.43, *χ*^2^: 44.13), and Elevated triglycerides (ln *OR*: -2.23, -log *P*: 1.47, *PRR*: 4.76, *χ*^2^: 5.45) (Figure 2, Table 2).

## Discussion

The “Clinical Guidelines for Proper Use of Hypnotics and Drug Withdrawal” of The Japanese Society of Sleep Research recommend the use of Z-drugs and not BZ drugs for sleep disorders 2. However, AEs have also been reported for Z-drugs, and these require attention. Therefore, for the purpose of identifying AEs to be noted when switching from BZ drugs to Z-drugs, a comparison between the AEs associated with both these drugs was conducted using volcano plots.

The results of volcano plots demonstrated that 12 AEs (HLTs) associated with Z-drugs showed a significant difference. Of these, six AEs (HLTs) were detected by *PRR*: Suicidal and self-injurious behaviour, Deliria, Parasomnias, Cortical dysfunction NEC, Psychiatric symptoms NEC, and Overdoses NEC; most of these were AEs related to the psychoneurotic system.

Of the six AEs (HLTs) that tended to occur with the use of Z-drugs and for which the safety signal was detected, Suicidal and self-injurious behaviour (Z-drugs *PRR*: 16.14 vs. BZ drugs *PRR*: 5.37), Deliria (Z-drugs *PRR*: 11.64 vs. BZ drugs *PRR*: 5.37), and Overdoses NEC (Z-drug *PRR*: 23.64 vs. BZ drugs *PRR*: 13.87) also occurred with the use of BZ drugs (with signal detection). The remaining three AEs (HLTs) Parasomnias, Cortical dysfunction NEC, and Psychiatric symptoms NEC were detected for Z-drugs. When switching from BZ drug to Z-drug as a hypnotic, it is essential to consider the AE characteristics of Z-drugs.

We noted that 20 AEs (HLTs) tended to occur with the onset of BZ drugs. Of these, five AEs (HLTs) were detected via *PRR*: Non-mechanical ileus, Ocular signs and symptoms NEC, Decreased physical activity levels, Myopathies, and Elevated triglycerides. Most of these were AEs related to physical symptoms.

Of the five AEs (HLTs) that tended to occur with the use of BZ drugs and for which the safety signal was detected, Ocular signs and symptoms NEC (Z-drugs *PRR*: 4.13 vs. BZ drugs *PRR*: 16.13) also occurred with the use of Z-drugs (with signal detection). The remaining four AEs (HLTs) were detected only for BZ drugs.

Thus, using volcano plots, the difference in the tendency of AE onset between the two drugs was clarified. This study revealed six AEs that should be noted when switching from BZ drugs to Z-drugs. Most of these were AEs related to the psychoneurotic system, which may be the caused because of the effect of Z-drugs on the brain.

Recently, using the U.S. Food and Drug Administration Adverse Event Reporting System, Harbourt *et al.* 17 reported the association of Z-drugs and complex sleep behaviors that result in serious injuries, including death.

The mechanisms by which Z-drugs cause complex sleep behaviors (*e.g.,* parasomnia) are not completely understood. Z-drugs selectively bind to the GABA alpha-1 subunit, which results in hypnotic effects without the anticonvulsant and myorelaxant effects associated with BZ drugs 18. The maintenance of slow-wave sleep may increase the likelihood of parasomnia induced by Z-drugs, especially compared with that induced by BZ drugs, owing to their less muscle-relaxing effect. In contrast to Z-drugs, BZ drugs reduce slow-wave sleep 19. These theories are also supported by the results of this study. Therefore, when switching from BZ drugs to Z-drugs, it is necessary to pay close attention to parasomnia.

Although Suicidal and self-injurious behaviour, Deliria, and Overdoses NEC showed a significant difference in Z-drugs, the safety signals were also detected with BZ drugs. Therefore, these AEs will continue to require caution when switching from BZ drugs to Z-drugs. The same is true for Ocular signs and symptoms NEC. Although Ocular signs and symptoms NEC showed a significant difference in BZ drugs, their signals were also detected with the Z-drugs.

“Benzodiazepine-induced ophthalmopathy” is recognized by some patients as dizziness, pain, and cloudiness 20. There have been several reports of this symptom since ancient times 20-24. In a recent study, Wakakura *et al.* 25 conducted a retrospective observational study involving a large population of patients from a single institution, and reported the need to remain aware of “benzodiazepine-induced ophthalmopathy” when prescribing etizolam, BZ drugs, and zolpidem (= Z-drug). Therefore, when switching from BZ to Z-drugs, it is essential to pay close attention to “benzodiazepine-induced ophthalmopathy” as Ocular signs and symptoms NEC.

In this study, we focused on the differences in the reporting ratio of AEs in Z-drugs and BZ drugs, and compared the trends of AE development. Therefore, if the reporting ratio is similar in AEs such that safety signals are detected for each drug, there is no significant difference in reporting ratio; therefore, it is not possible to clarify the difference in expression tendency. For such AEs in which the reporting ratio is similar for each drug, it is considered necessary to construct an analysis model focusing on the onset timing rather than a frequency-theoretic statistical model. For the proper use of hypnotics, research focusing on the difference in the onset timing of hypnotics is desired in the future.

## Limitation

There were several limitations to this study. JADER is a case report database of AEs, and the total number of patients using this drug is not known, thereby preventing the calculation of incidence and prevalence. Therefore, the comparison between the Z-drugs and the BZ drugs made in this study is a comparison of reporting ratios rather than a comparison of incidence rates.

Although differences in reporting ratios are often used to detect the safety signals of AEs as a disproportionality analysis, the detected safety signals are known to be subject to reporting bias (*e.g.,* Weber effect 26, notoriety effect 27, and ripple effect 27). Naturally, these reporting biases are also unavoidable in the volcano plot analysis, as these biases are attributable to the case series when the database (JADER) was created.

## Conclusion

In this study, to understand the AEs that should be noted when switching from BZ drugs to Z-drugs, the differences between the AEs that onset with BZ drugs and Z-drugs were evaluated using volcano plots and safety signals using JADER. The volcano plot revealed 12 characteristic Z-drug-induced AEs. Of these, there were six AEs for which signals were detected. Parasomnias, Cortical dysfunction NEC, and Psychiatric symptoms NEC were detected only in Z-drugs, safety signals of Suicidal and self-injurious behaviour, Deliria, and Overdoses NEC were also detected in BZ drugs, but the safety signal strength was much higher with the Z-drug. Therefore, attention should be paid to the onset of these AEs when switching from BZ drugs to Z-drugs. Understanding the difference between these Z-drug- and BZ drug-related AEs is important for the proper use of hypnotics.

## Figures and Tables

**Table 1:** Target suspected drugs

Z-drugs	BZ drugs
Eszopiclone Zopiclone Zolpidem	Triazolam Brotizolam lormetazepam rilmazafone

**Table 2:** Adverse events with significantly different onset, and their signal scores.

				
		Volcano plot		Z-drug	BZ drug
Class	Adverse Event (High Level Term)	ln OR	*-*log* P*	*n*_11_, PRR, χ^2^ (PRR 95%CI)	*n*_21_, PRR, χ^2^ (PRR 95%CI)
Z-drug	Suicidal and self-injurious behaviour ^*†^	1.10	13.84	208, 16.14, 2743.94 (14.09-18.48)	55, 5.37,188.70 (4.13-6.99)
	Deliria ^*†^	0.88	6.42	130, 11.64, 1193.14 (9.79-13.83)	42, 4.82, 121.77 (3.56-6.52)
	Parasomnias ^*†^	3.28	4.34	17, 23.47, 309.27 (14.24-38.69)	NA
	Cortical dysfunction NEC ^*^	2.76	2.18	10, 3.62, 16.14 (1.94-6.75)	NA
	Psychiatric symptoms NEC ^*^	2.66	2.18	9, 2.51, 6.63 (1.30-4.83)	NA
	Overdoses NEC ^*†^	0.49	2.09	92, 23.64, 1776.82 (19.13-29.23)	43, 13.87, 477.76 (10.24-18.77)
	Neurological signs and symptoms NEC	0.82	1.98	39, 1.14, 0.53 (0.83-1.56)	13, 0.50, 5.98 (0.29-0.86)
	Pulmonary embolism and thrombosis	1.51	1.50	12, 0.98, 0.004 (0.55-1.72)	2, 0.22, 4.97 (0.05-0.86)
	Breast and nipple neoplasms malignant	2.12	1.39	5, 1.25, 0.06 (0.52-3.02)	NA
	Cholecystitis and cholelithiasis	2.12	1.39	5, 0.73,0.25 (0.31-1.77)	NA
	Disturbances in initiating and maintaining sleep	2.12	1.39	5, 1.62, 0.65 (0.67-3.91)	NA
	Vascular hypotensive disorders	1.11	1.38	16, 0.33, 21.73 (0.20-0.53)	4, 0.11, 28.80 (0.04-0.29)
BZ drug	Bullous conditions	-0.74	3.70	41, 0.53, 17.03 (0.39-0.72)	64, 1.10, 0.52 (0.87-1.40)
	Non-mechanical ileus ^†^	-3.33	3.68	NA	10, 3.53, 15.50 (1.89-6.57)
	Ocular signs and symptoms NEC ^*†^	-1.33	2.32	6, 4.13, 11.05 (1.84-9.25)	17, 16.13, 214.17 (9.91-26.25)
	Urinary abnormalities	-2.85	2.20	NA	6, 0.95, 0.004 (0.42-2.10)
	Cholestasis and jaundice	-1.47	2.20	4, 0.27, 7.27 (0.10-0.72)	13, 1.17, 0.16 (0.68-2.01)
	Decreased physical activity levels ^†^	-2.68	2.20	NA	5, 11.06, 34.84 (4.53-27.00)
	Myopathies ^†^	-0.57	2.20	41, 1.38, 4.02 (1.02-1.88)	54, 2.43, 44.13 (1.87-3.16)
	Hepatic enzymes and function abnormalities	-0.43	1.83	65, 0.78, 3.91 (0.61-0.99)	74, 1.18,1.94 (0.94-1.48)
	Allergies to foods, food additives, drugs and other chemicals	-0.58	1.69	30, 0.32, 43.21 (0.23-0.46)	40, 0.57, 12.66 (0.42-0.78)
	Muscular autoimmune disorders	-2.48	1.47	NA	4, 1.90, 0.92 (0.71-5.08)
	Reproductive tract signs and symptoms NEC	-2.48	1.47	NA	4, 1.24, 0.02 (0.46-3.30)
	Gait disturbances	-2.48	1.47	NA	4, 0.79, 0.06 (0.30-2.11)
	Adenoviral infections	-2.23	1.47	NA	3, 3.37,2.87 (1.08-10.51)
	Elevated triglycerides ^†^	-2.23	1.47	NA	3, 4.76, 5.45 (1.52-14.89)
	Infections NEC	-2.23	1.47	NA	3, 0.23, 7.05 (0.07-0.71)
	Bacterial infections NEC	-2.23	1.47	NA	3, 0.16, 12.14 (0.05-0.51)
	Muscle tone abnormalities	-2.23	1.47	NA	3, 0.21,8.15 (0.07-0.65)
	Schizophrenia NEC	-2.23	1.47	NA)	3, 2.84, 1.94 (0.91-8.84)
	Site specific necrosis and vascular insufficiency NEC	-2.23	1.47	NA	3, 3.30, 2.75 (1.06-10.31)
	Rashes, eruptions and exanthems NEC	-0.86	1.37	9, 0.20, 28.73 (0.10-0.38)	16, 0.47, 9.17 (0.29-0.77)
